# Phylogeographic structure of common sage (*Salvia officinalis* L.) reveals microrefugia throughout the Balkans and colonizations of the Apennines

**DOI:** 10.1038/s41598-022-20055-4

**Published:** 2022-09-21

**Authors:** Ivan Radosavljević, Zlatko Satovic, Romeo di Pietro, Marija Jug Dujaković, Filip Varga, Danijel Škrtić, Zlatko Liber

**Affiliations:** 1 Division of Botany, Department of Biology, University of Zagreb, Faculty of Science, Marulićev trg 9A, HR, 10000 Zagreb, Croatia; 2 Centre of Excellence for Biodiversity and Molecular Plant Breeding (CroP-BioDiv), Svetošimunska Cesta 25, HR, 10000 Zagreb, Croatia; 3 Department of Seed Science and Technology, University of Zagreb, Faculty of Agriculture, Svetošimunska Cesta 25, HR, 10000 Zagreb, Croatia; 4 Department PDTA (Section Environment and Landscape), Sapienza” University of Rome, Via Flaminia 72, 00196 Rome, Italy; 5 Department for Plant Sciences, Institute for Adriatic Crops and Karst Reclamation, Put Duilova 11, 21000 Split, Croatia

**Keywords:** Population genetics, Haplotypes, Molecular evolution, Population genetics

## Abstract

Studying the population-genetic and phylogeographic structures of a representative species of a particular geographical region can not only provide us with information regarding its evolutionary history, but also improve our understanding of the evolutionary processes underlying the patterns of species diversity in that area. By analysing eight highly polymorphic microsatellite loci and two chloroplast DNA regions, we have investigated the influence of Pleistocene climate fluctuations on the evolutionary history of *Salvia officinalis* L. (common sage). The populations with the highest genetic diversity were located in the central parts of the Balkan distribution range. A large group of closely related haplotypes was distributed throughout the Balkans and the central Apennines, while the private lineage occupied the southern Apennines. In addition, two highly differentiated lineages were scattered only over the Balkans. The results suggest that a single refugium of the studied species from the last glacial period was located in the central part of the range in the Balkans. Numerous microrefugia, probably spanning several glaciation cycles, were scattered across the Balkans, while colonisation of the Apennines from the Balkans occurred at least on two occasions.

## Introduction

During the Quaternary period, the global climate was characterized by pronounced global temperature fluctuations. Periodical glaciations across the Northern Hemisphere became regular occurrences^1^, strongly influencing both the genetic diversity and phylogeographical patterns of numerous European species^2–5^. During climatic oscillations, the distribution ranges of many species exhibited substantial fluctuations due to changes in various climatic factors^6,7^ as well as sea level changes linked to global temperature^8–10^. Sea level oscillations were especially important in areas surrounding shallow seas, where they were responsible for the opening and later closing of land bridges between geographically isolated landmasses^11^.

According to Medail and Diadema^7^, the refugium is “an area where distinct genetic lineages have persisted through a series of Tertiary or Quaternary climate fluctuations owing to special, buffering environmental characteristics”. Comparative phylogeographic analyses of numerous plant and animal species recognized three northern Mediterranean peninsulas as major European refugia^11–14^. Although Hewitt^14^ suggested that each of the three peninsulas should be treated as a single refugium, the “refugia-within-refugia” model^15^ seemed more appropriate on numerous occasions^16–22^. The model suggests that after climate conditions became favourable, geographically scattered lineages that survived in isolated locations would reclaim the previous distribution range of the species, while the genetic fingerprints of glacial isolation would still be visible. Refugial populations are expected to be characterized by a specific molecular signature, such as the presence of unique yet highly divergent haplotypes that emerge as a consequence of long-term isolation through multiple glacial cycles^15,22,23^. The level of geographic structuring of the lineages can provide information regarding the expansion following the occurrence of favourable environmental conditions. If lineages are spatially structured, recolonization from localized refugia likely occurred, enabling the geographical expansion of the divergent haplotype. However, highly localized haplotypes of divergent lineages scattered throughout the distribution area of studied taxa indicate their pulsating range distribution, with repeating cycles of spatially localized expansions and contractions that did not increase their distribution area^22^.

From the inability to explain the postglacial expansion of *Fagus grandifolia* in North America based on the known dispersal mechanisms of the species (i.e., Reid’s Paradox^24^), the concept of microrefugia was borne^25^. The term “microrefugia” was first introduced by Rull et al.^26^, and it is now widely accepted and used in the context of postglacial colonization. It suggests that recolonization did not occur through a single large-scale expansion event from an area (i.e., refugia) where species survived the period of unfavourable climatic conditions. Instead, recolonization occurred simultaneously from multiple areas “with local favourable environmental features, in which small populations can survive…protected from the unfavourable regional environmental conditions”^27^.

The Adriatic Sea is located in the northern Mediterranean between the Apennines and the Balkans. Its northern and central sections are rather shallow, while the southern section is characterized by a depression more than 1200 m deep^28^. Consequently, during the last glacial maximum (LGM), most of today’s northern and central Adriatics were land connecting two peninsulas, while the sea remained only in the southern parts^29,30^. Such geographic rearrangements have strongly influenced the ranges and phylogeographic structures of numerous amphi-adriatic species or groups of closely related species, of which there is ample empirical evidence^19,31–35^. The majority of these studies suggested that the expansion occurred from the Balkans to the Apennines, and only on rare occasions did the results suggest expansion in the opposite direction^36^.

Investigation of population-genetic and phylogeographic structures of a representative species in a specific habitat or geographic area can not only provide us with information regarding the species’ evolutionary history, but it also improve our understanding of evolutionary processes that underlie biodiversity patterns in the studied area. Common sage (*Salvia officinalis* L., 1753) is an endemic species of the northern Mediterranean and one of the most common species within its distribution area. It grows on shallow limestone soils^37^, which make up most of the western coastal region of the Balkans^38,39^. However, it covers substantially smaller portions of the Apennines^40^, which reflects the species distribution. In the Balkans, it grows abundantly from the Gulf of Trieste in the north to central Greece in the south^41^, while fewer and localized populations are present in the central and southern parts of the Apennine Peninsula^42–45^. Until now, comprehensive population genetic and phylogeographic analyses that would cover the entire distribution area of the species have not been performed. Rešetnik et al.^46^ performed an SSR-based population genetic analysis of a limited number of populations from the Balkan Peninsula, with emphasis on the detection of naturalized populations throughout the area. Recently, Jug-Dujaković et al.^47^ analysed populations from Croatia, thus covering only a fraction of the species distribution area.

By conducting the analysis of highly polymorphic microsatellite loci and two chloroplast DNA regions on the sample set covering the entire distribution range of the species, we aimed to reconstruct past demographic oscillations that shaped the current genetic structure of the species. We tried to answer additional questions with this research: Is it possible to detect the refugial areas of *Salvia officinalis* L. during the LGM and more ancient ones that stretched across multiple glaciation cycles? Will the phylogeographic analysis support the “refugia within refugia” hypothesis? Is it possible to confirm the “out of Balkans” hypothesis?

## Results

### Microsatellite analysis

**Figure 1:**
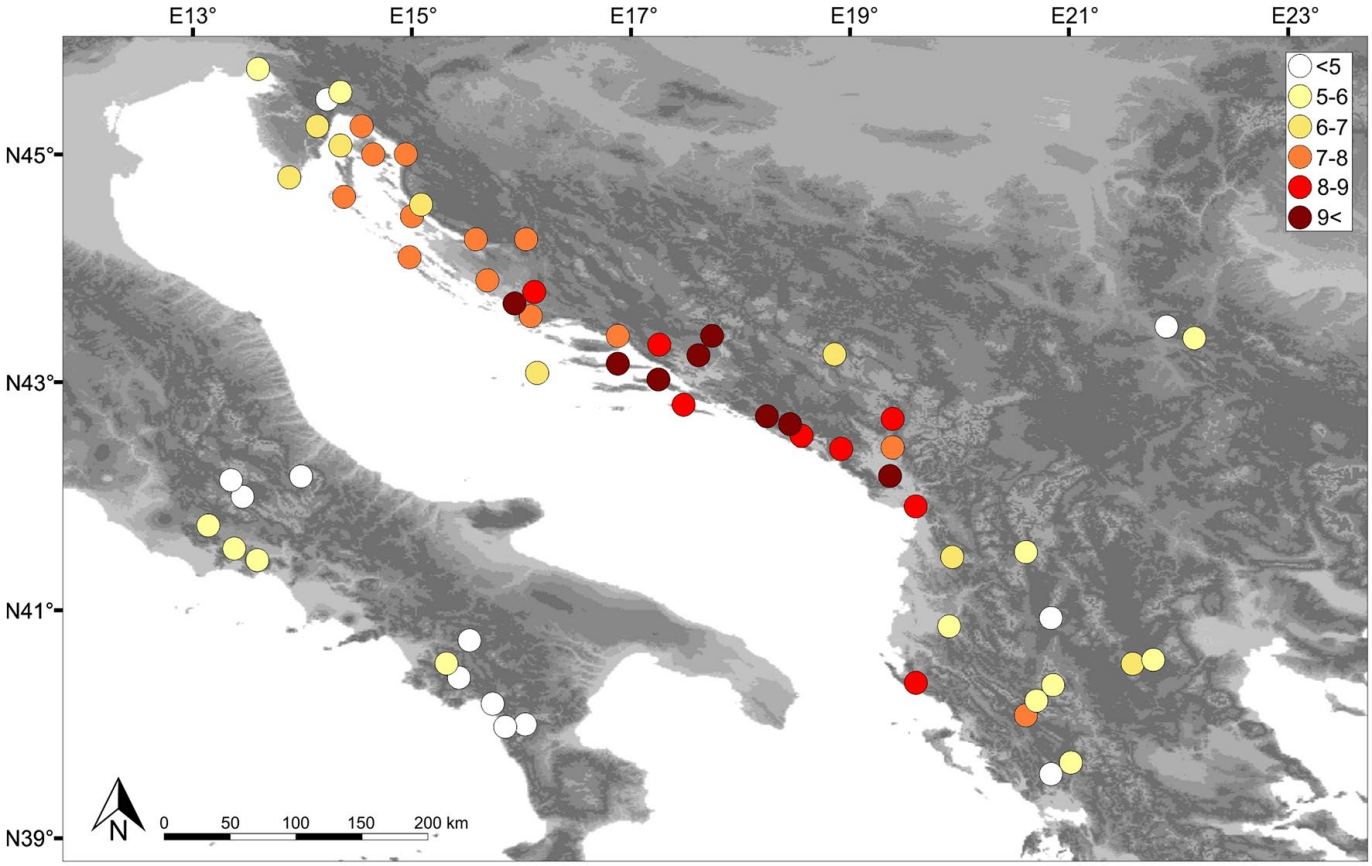
Distribution of allelic richness among the studied common sage (*Salvia officinalis L.*) populations as revealed by microsatellite markers. Coloured dots represent analysed populations of *S. officinalis*. Colours, from white to dark red, indicate allelic richness levels for individual populations. Allelic richness values for each colour are presented in the upper-right corner. The satellite imagery was obtained from Natural Earth public domain map dataset (https://www.naturalearthdata.com/downloads/10m-raster-data/10m-gray-earth/). CorelDraw Graphics Suite X7 Version 17.1.0.572 (Corel Corp., Ottawa, Canada) was used to create the figure.

PCR amplification was successful in 1350 samples that were used for further analyses. The population genetic parameters obtained for each population are shown in Supplementary Table S1. The highest values of heterozygosity levels and allelic richness were detected in populations located along the central and southern parts of the eastern Adriatic coast. In more marginal parts of the distribution area in the Balkans and especially throughout the Apennines, populations were characterized by substantially lower values of these parameters (Fig. 1). The same pattern was observed with the occurrence of private alleles, as the vast majority were detected in the central group of populations from the eastern Adriatic coast.

**Figure 2:**
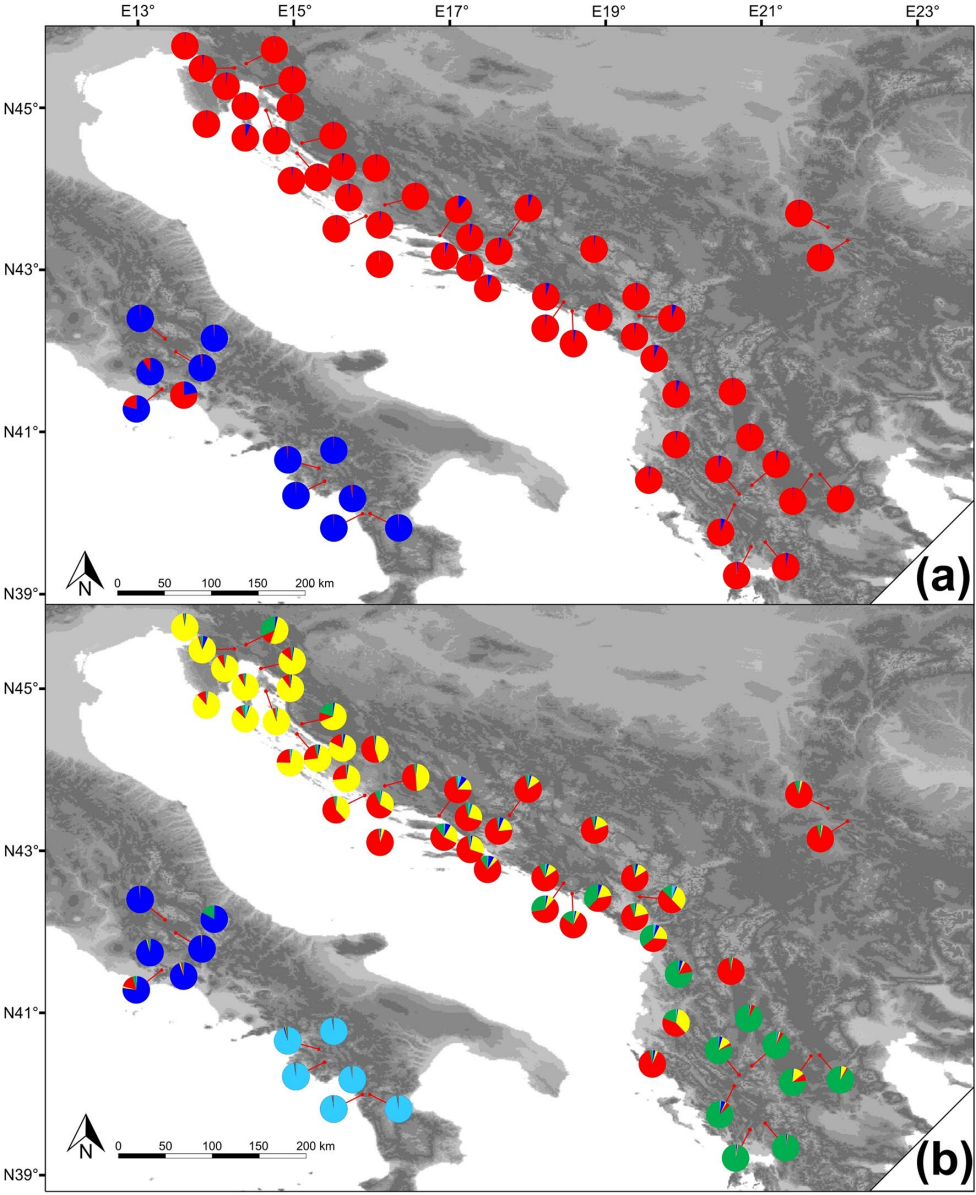
Microsatellite-based genetic structure of common sage (*Salvia officinalis* L.) derived from Bayesian model-based clustering analysis using STRUCTURE. (**a**) Population structure assuming K = 2 and (**b**) K = 5. Each colour represents one genetic cluster: light blue—SAp, dark blue—CAp, yellow—NBalk, red—CBalk, and green—SBalk. The satellite imagery was obtained from Natural Earth public domain map dataset (https://www.naturalearthdata.com/downloads/10m-raster-data/10m-gray-earth/). CorelDraw Graphics Suite X7 Version 17.1.0.572 (Corel Corp., Ottawa, Canada) was used to create the figure.

In the STRUCTURE and subsequent likelihood value analyses, by far, the highest ΔK value was obtained for K = 2 (ΔK = 4889.88), suggesting the presence of two well-differentiated genetic groups, each located on one side of the Adriatic Sea (Fig. 2a). The following highest ΔK value was present at K = 5, suggesting the presence of a higher-resolution genetic structure throughout the species distribution range (Supplementary Table S1, Fig. 2b). It is worth noting that at K = 2, the vast majority of populations were characterized by very low admixture levels. However, for at least some of the populations from the central Apennines, this was not the case, and one of them (P08) was even grouped together with Balkans populations (Fig. 2a). At K = 5, two genetic clusters were observed in the Apennines, one in the southern parts (SAp) and the other in the central parts (CAp). At the same time, three geographically well-defined clusters were recognized in the Balkans, in the northern and central parts of the western Balkans coastal region (NBalk and CBalk, respectively), and in the southern Balkans (SBalk). After obtaining clustering results, basic population genetic parameters were again calculated for each of the five genetic groups, and the results are summarized in Table 1. The results highlighted the strong contrast between the clusters, with the CBalk cluster exhibiting by far the highest values of all the parameters, especially the number of private alleles. In contrast, the CAp cluster was on the opposite side of the spectrum, without a single private allele and with depleted levels of genetic variability.

**Table 1:** Population genetic parameters of five *S. officinalis* genetic clusters as recognized by Bayesian model-based clustering analysis using STRUCTURE.

Genetic cluster (K = 5)	N_pop_	*N*_ar_	*N*_pr_	*N*_par_	*H*_O_	*H*_E_
SAp	6	4.562	6	0.88	0.562	0.558
CAp	6	3.997	0	0.15	0.496	0.490
NBalk	15	6.678	2	0.48	0.696	0.711
CBalk	26	8.039	26	1.92	0.718	0.749
SBalk	9	5.881	9	1.39	0.639	0.657

Genetic cluster (K = 5)—genetic clusters as recognized by STRUCTURE software at K = 5. SAp, CAp, NBalk, CBalk, SBalk—clusters occupying the southern Apennines, central Apennines, northern parts of the western Balkans coastal region, central and southern parts of the western Balkans coastal regions including inland populations, and the southern Balkan Peninsula, respectively.

*Lat* latitude, *Long* longitude, *Npop* number of populations included, *N*_*ar*_ allelic richness, *N*_*pr*_ number of private alleles, *N*_*par*_ private allelic richness, *H*_*O*_ observed heterozygosity, *H*_*E*_ expected heterozygosity.

For the DIYABC analysis, the first scenario had the highest posterior probability (Supplementary Table S2), which suggested that the cluster from the Southern Apennines was the first to split from the remaining ancestral group, which later gave birth to four other clusters. The divergence times for the first and second splits were estimated to be 7020 (t2) and 582 generations (t1), respectively, suggesting prolonged isolation of the SAp cluster. However, it should be noted that 95% confidence intervals for both results were wide (1330–64,500 and 131–2060, respectively), making their interpretation speculative. The estimated effective population size of clusters ranged from 945 (cluster CAp) to 9140 (cluster CBalk) (Supplementary Table S3).

Model checking provided further evidence of the validity of Scenario 1, since the observed dataset fell within the cluster of points of the simulated datasets obtained based on the posterior distribution (Supplementary Fig. S1).

### Chloroplast DNA sequence analysis

**Figure 3:**
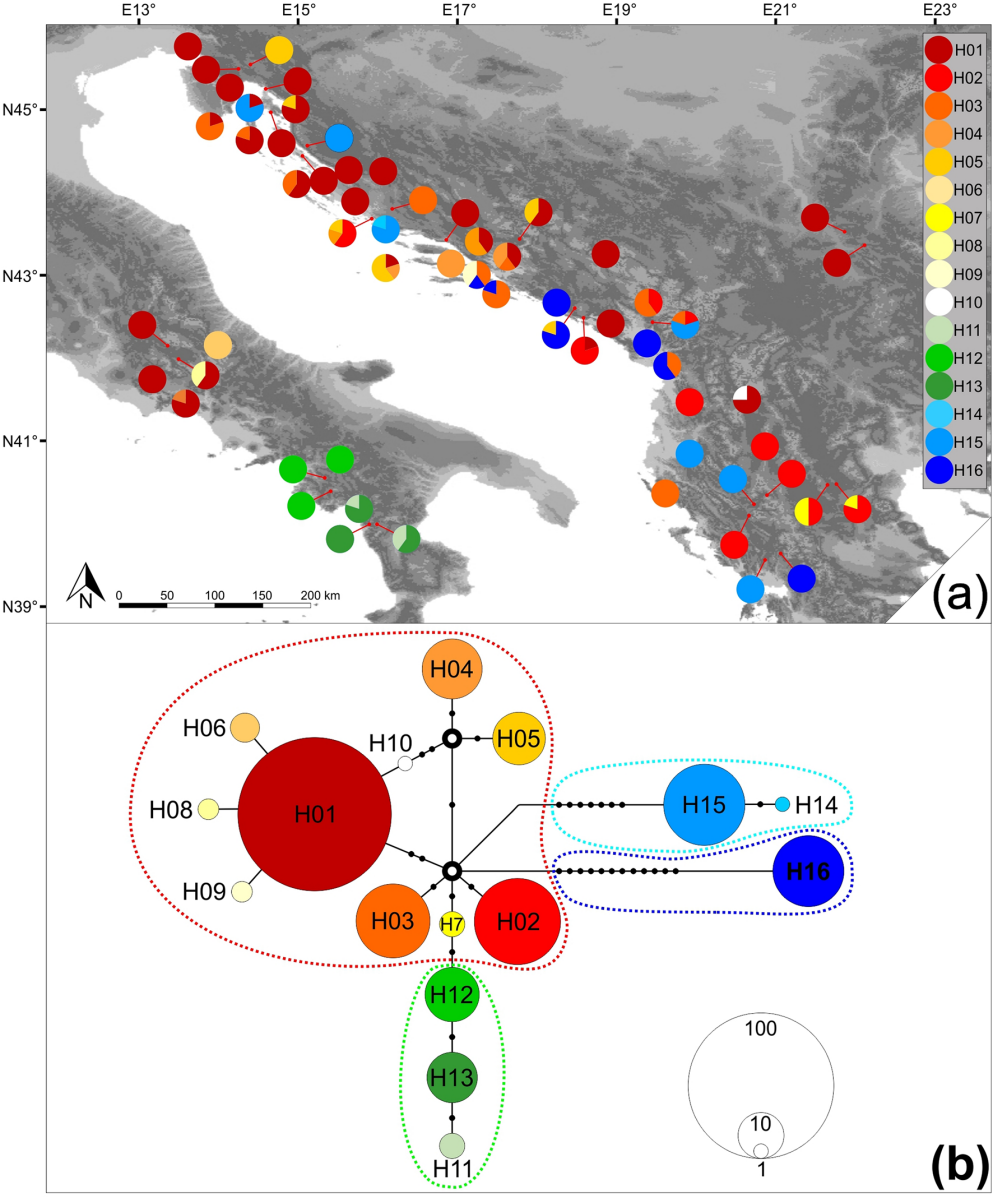
(**a**) Geographic distribution of 16 cpDNA haplotypes throughout common sage (*Salvia officinalis* L.) distribution range. The proportion of colours in each pie corresponds to detected haplotype frequencies in the population. The haplotype colour code is on the right. The satellite imagery was obtained from Natural Earth public domain map dataset (https://www.naturalearthdata.com/downloads/10m-raster-data/10m-gray-earth/). CorelDraw Graphics Suite X7 Version 17.1.0.572 (Corel Corp., Ottawa, Canada) was used to create the figure. (**b**) Median-joining haplotype network. Each filled circle indicates a unique haplotype, and black dots represent mutational steps (i.e., missing haplotypes). Two ancestral haplotypes are presented as empty bold circles in the middle of the network. The diameter of circles is proportional to the observed overall frequency of the haplotype, with a corresponding scale in the lower right corner. Dashed lines indicate four haplotype groups: the red line the largest group distributed throughout the Balkans and the Central Apennines, the green line one from the Southern Apennines, and light blue and dark blue lines indicate two highly differentiated lineages from the Balkans.

The sequencing of selected cpDNA regions was successfully performed in 300 individuals. In six populations, DNA samples from four individuals were successfully used in the analysis, while in the 55 populations, all five samples were amplified and sequenced. We were unable to successfully process samples from one population (P08 from the Central Apennines) even after repeated attempts. Sixteen haplotypes were identified by the analysis (Fig. 3a and Supplementary Table S4), and the constructed haplotype network revealed four groups of haplotypes (Fig. 3b). The largest one comprises ten haplotypes (H01–H10), characterized by low levels of differentiation, with typically only one or two mutation steps between neighbouring haplotypes. The majority of these haplotypes were not specific to a certain geographic area. The group spread throughout the Balkans and the central Apennines. Then, there was a group of three haplotypes from the Southern Apennines (H11, H12, and H13). This was the only group with a pronounced geographic structure, and its private haplotypes were unique to the region. Finally, in the Balkans, two highly differentiated lineages were detected, with haplotypes H14 and H15 presenting the first and H16 as the sole representative of the second. Populations harbouring the H14 and H15 haplotypes were scattered across the western Balkans, while the H16 haplotype was found across the central and southern parts, without any recognizable geographical structure.

Haplotype diversity levels quantified as unbiased haplotype diversity (*H*d) and nucleotide diversity (π) were calculated for the genetic clusters recognized by STRUCTURE. The highest values of both parameters were found in the CBalk group of populations (0.811 and 0.00405, respectively), followed by the SBalk group (0.757 and 0.00434, respectively). In the remaining groups (SAp, CAp and NBalk), moderate values of *H*_d_ were found (0.606, 0.482, and 0.486, respectively), while the lowest π values were detected in the NBalk (0.002001) and Apennines groups (0.00048 in SAp and 0.00058 in the CAp).

The presence of phylogenetic structure was tested by comparing two estimates of genetic variation: *G*_ST_ and *N*_ST_. The *N*_ST_ value (0.814) was significantly higher (P < 0.01) than the *G*_ST_ value (0.733), thus reflecting the fact that distinct haplotypes mixed in the same population were, on average, more closely related than distinct haplotypes from different populations.

No signs of severe recent population expansion or bottleneck were detected, as estimates of both Tajima's *D* and Fu's *F*_S_ were nonsignificant (− 0.142 (P = 0.527) and 3.119 (P = 0.831), respectively).

### Environmental niche modelling

**Figure 4:**
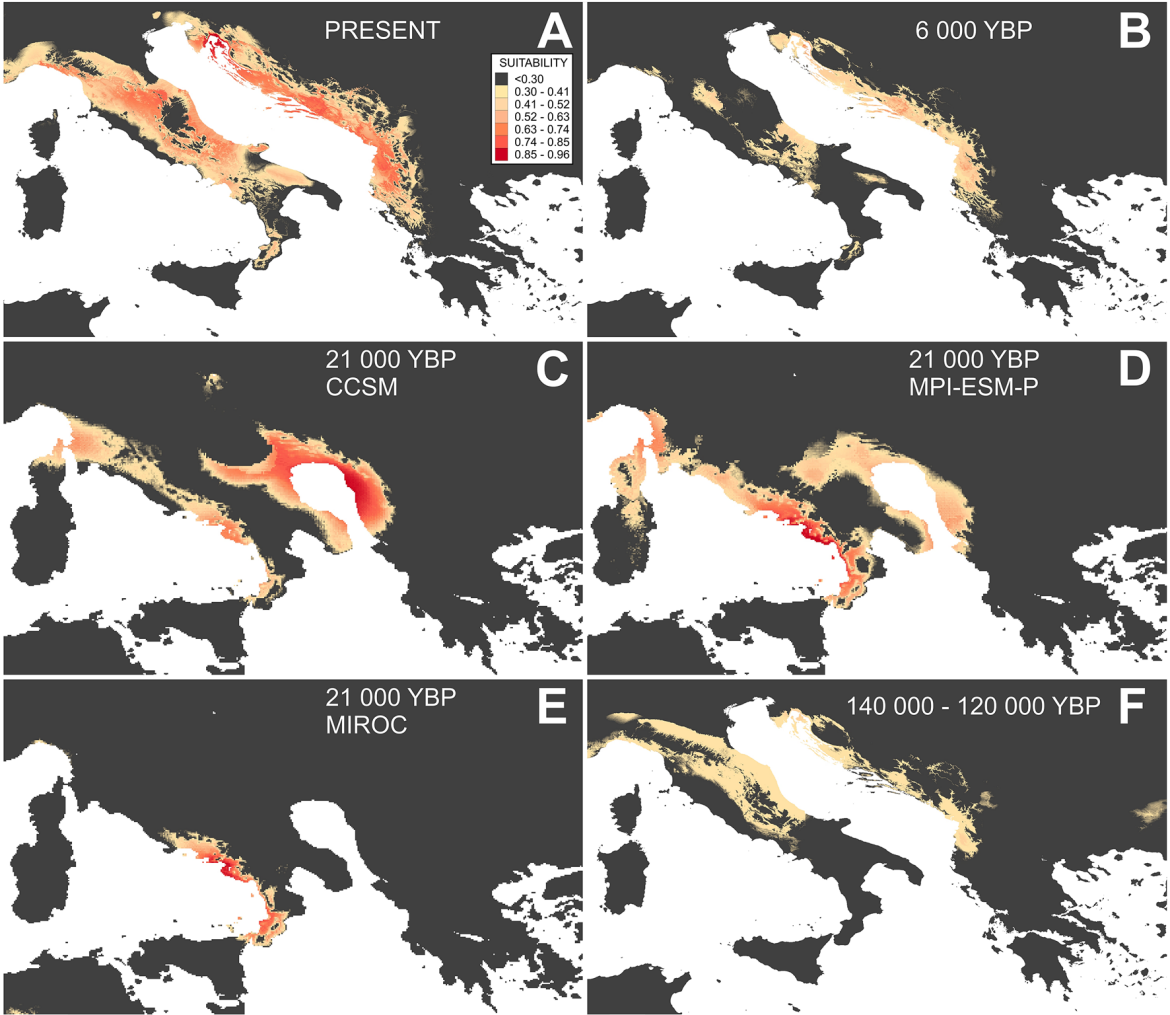
Environmental suitability map for common sage (*Salvia officinalis* L.) based on ecological-niche modelling for different periods: (**a**) present, (**b**) mid-Holocene, (**c**) Last Glacial Maximum CCSM, (**d**) Last Glacial Maximum MPI-ESM-P, (**e**) Last Glacial Maximum MIROC, and (**f**) last interglacial conditions. The colour code indicates habitat suitability, from light yellow (low suitability) to dark red (high suitability). The colour code for all figures is placed on the right side of figure (**a**).

Based on the results of the multicollinearity test, 13 bioclimatic variables were excluded from further analysis, while the remaining six variables (BIO1—annual mean temperature, BIO3—isothermality (BIO2/BIO7) (× 100), BIO4—temperature seasonality (standard deviation × 100), BIO8—mean temperature of wettest quarter, BIO9—mean temperature of driest quarter, BIO14—precipitation of driest month) were used. Their relative contributions to the niche model were 7, 0.1, 19.4, 20.1, 23.4 and 30.1%, respectively. The model characterized by the smallest AICc value (i.e., the one with Maxent’s regularization multiplier = 2) was selected. The model obtained for the present time was consistent with the distribution area of the species in the Balkans, while in the Apennines, this was not the case, as the suitable area suggested by the model was substantially larger (Fig. 4a). For the LGM period (21,000 years before present (YBP)), the CCSM (Fig. 4c) and MPI-ESM-P (Fig. 4d) models yielded similar results: the suitable area was restricted to the coastal area of the paleo-Adriatic Sea, corresponding to today’s central and southern Adriatic. The land bridge between peninsulas and parts of the middle and southern Apennines was environmentally suitable for the existence of the species as well. The results of the MIROC model for the LGM (Fig. 4e) seem highly unlikely, as the model characterized the entire Balkan Peninsula as highly unsupportive for the common sage existence during the LGM, but at the same time recognized the narrow coastal area in the southern and middle Apennines as highly suitable. Finally, modelling results for the mid-Holocene (6000 YBP) (Fig. 4b) and last interglacial period (140,000–120,000 YBP) (Fig. 4f) were very similar. The eastern Adriatic coastal region and middle parts of the Apennines were recognized to be only moderately suitable, while the southern Apennines were apparently environmentally unsuitable for the existence of the common sage.

## Discussion

To analyse past demographic oscillations of the common sage through as wide a period as possible, we used microsatellites and cpDNA sequences. Characterized by exceptionally high mutation rates, microsatellites are well-suited for analyses of the recent demographic events that shaped the genetic structure of the species^48^. At the same time, cpDNA sequences are a standard tool for phylogeographic analyses known for their low mutation rates^49^ thus identifying more ancient events. Therefore, we discuss the two sets of results separately.

The population genetic analysis revealed strong contrasts in genetic richness levels among populations from different geographic regions. Similar to *Edraianthus graminifolius*^32^, most of the Balkan populations of common sage were characterized by substantially higher values of allelic richness than Apennine populations. In addition, populations from the central part of the Balkan distribution area showed significantly higher allelic richness and private allele values than any other group of populations. During rapid colonization, newly emerging populations have progressively depleted levels of genetic variability as they are founded more away from the core populations^50^, so this is likely the most plausible explanation for the obtained results. Such a finding strongly supports the possibility of this area being the species refugium during the LGM. Since multiple refugial areas were not recognized, the "refugia within refugia" concept^15^ seems inappropriate for this instance, at least on the microsatellite time scale.

The STRUCTURE results provided evidence of clear geographic structuring at different resolution levels. As the Adriatic Sea is the most prominent biogeographic barrier in the region, it was not unexpected to see two clusters (at K = 2) occupying neighbouring peninsulas. At a higher resolution level (K = 5), spatial structuring remains clear, with clustering of the populations according to their geographical positions. In contrast to other studies of species occurring throughout the western Balkans^20,51,52^, no clear geographic barriers were recognized between clusters. Consequently, in the contact zones, admixed populations were present because the interpopulation gene flow constantly blurs differentiation among clusters. However, there is a slight possibility that the Balkan's genetic structuring emerged as an artefact of the STRUCTURE algorithm. This is especially likely when populations of highly contrasting genetic diversity levels are included in the analysis^53^ (Table 1).

In the Apennines, some populations from the central parts were characterized by admixed structures, with various proportions of the Balkan clusters’ genetic material included. Thus, the recent gene flow with the Balkan populations seems obvious. In contrast, populations from the southern Apennines retained genetic purity, suggesting their prolonged isolation (Fig. 2b). These conclusions are also supported by the DIYABC analysis. The generation time of this perennial species is unknown, but from the obtained results, it can be estimated. We can assume that the central Apennines cluster separated from the Balkan clusters after the LGM ended and that the rising sea separated the Balkans from the Apennines. Since this event took place 15,000–10,000 YBP^54^, based on the obtained number of ca. 580 generations that have passed since then, we can assume that the common sage generation time ranges from 17 to 26 years. Based on this value, we can further estimate the earlier divergence time (t2) ca. 180,000–119,000 YBP, which corresponds with the time of the penultimate glacial maximum that lasted from 194,000 to 135,000 YBP^55^ and the following last interglacial, which peaked approximately 120,000 YBP^56^. Such conclusions are in agreement with both SSR and cpDNA results, which suggested that the split of the southern Apennines populations occurred sometime before the central Apennines cluster split from the Balkan populations. Although the results undoubtedly support the colonization of the Apennines on two separate occasions, we should be cautious when assessing the time scale of these events since only medians of the generation numbers were taken into account here. Confidence intervals of the estimated values were wide, making the conclusions based on these results speculative.

The obtained ENM results confirmed that the land bridge that stretched across the middle parts of today's Adriatic Sea^9,29^ was likely characterized by suitable climatic conditions for species survival. It seems that during the LGM, the proximity of seawater masses and the resulting temperature-buffering effect could be of great importance for sustaining the species along the paleo-sea coastline^52,57^. For the northern Adriatic and southern Balkans regions, modelling suggests that, in contrast to the central parts of the Balkans, during the LGM, they were unsuitable for species survival, at least on large geographic scales. Consequently, it seems likely that the common sage experienced a severe contraction in its distribution range during the last glaciation, followed by range expansion when the conditions became more favourable. At this point, it is worth noting that ENM for the present time gave very accurate results for the Balkans but not the Apennines, likely because the patchiness of the limestone soil throughout the Apennines^40^ is a limiting factor for the expansion of this species. Such a result serves as a reminder that the actual distribution of the species is influenced by numerous biotic and abiotic elements, and bioclimatic variables represents just a fraction of them.

Numerous studies have already investigated various Balkan and amphi-Adriatic taxa in search of genetic fingerprints of past demographic oscillations. However, the majority of these studies were either focused on species occupying mountain or inland areas^32,58,59^ or were focused solely on the phylogeography of the studied species^19,33–35,60^. Nonetheless, there are a few available studies comparable to ours in which taxa with similar distribution ranges and ecological preferences were analysed. Kučera et al.^51^ addressed the population-genetic structure of *Cardamine maritima* (Brassicaceae), and the results suggested that the more ancient populations were detected in specific habitats (open mountain slopes and gorges oriented towards the sea) and not any specific geographic region. Grdiša et al.^61^ presented results from the population-genetic analysis of *Tanacetum cinerariifolium* (Asteraceae), which also grows throughout western parts of the Balkan Peninsula. The results were in contrast to ours, as the northern Adriatic region was suggested as the likely LGM refugium for the species. Finally, the results from Surina et al.^20^ were more similar to ours, as higher gene diversity and rarity index values were detected in southern populations of *Edraianthus tenuifolius* (Campanulaceae) in the same region where highly diversified populations of common sage were recognized.

Barely any similarities in patterns between cpDNA and SSR results were detected. The most prominent feature of the haplotype network was the existence of two highly differentiated lineages distributed along the eastern coast of the Adriatic Sea without any representatives in the Apennines. Without a doubt, populations that harbour these haplotypes are of ancient origin (in further text: ancient populations). In contrast to similar studies^22,32,62^, these lineages are not geographically structured, which blurs their role during recolonization. Nonetheless, their locations, which likely sheltered them throughout multiple glaciation cycles, represent the species microrefugia. Meanwhile, the absence of these haplotypes from the Apennines implies that the species was much earlier present in the Balkans than in the Apennines.

Since diverged haplotypes were distributed across large geographic areas and were found in many populations, it is obvious that they did not evolve numerous times in situ. Sometime during the species evolutionary history, they were substantially more abundant than they are today, occupying larger parts of the paleo-distribution area. Over time, unfavourable conditions and perhaps other lineages suppressed them, and they became quarantined in scattered microrefugia. In general, it is assumed that microrefugia played an important role during postglacial expansions^63–65^ and that Pleistocene recolonization patterns can hardly be explained without them^27^. However, such assumptions were not supported by our results. If these populations had such a role during the postglacial colonization, their haplotypes would be, if not more abundant than they are, geographically structured, which they are not. Did ancient populations play a stimulating role during the expansion of other lineages through genetic support by sharing genes of importance for local adaptation? Is it possible that these diverged haplotypes represent once-dominant ecotypes well adapted to the colder climate that was dominant throughout much of the Pleistocene^27^? Or are they the remnants of extinct and closely related species assimilated by a more abundant species, the common sage? From the obtained results, it is impossible to answer any of these questions. On some occasions, similar phylogeographic results were explained as evidence of cryptic species' existence^66,67^. However, at least some support for such a conclusion from the nuclear-genome results is expected^68–71^. In contrast to plastid genomes of ancient populations, their nuclear genomes do not stand out in any aspect compared to those of surrounding populations. Since plastids are inherited maternally^72^ and SSRs biparentally, it is not a surprise to see such divergence between plastid and nuclear genomes. The nuclear-level genetic signature that would possibly distinguish ancient populations from the others was likely washed away by intense and persistent pollen-mediated gene flow. At the same time, the nonrecombinant plastid genome inherited via species-specific barochory-type seeds^73^ was retained in numerous microlocations across today's distribution range.

During a prolonged period that possibly spanned over multiple glaciation cycles, the majority of the populations harbouring haplotypes closely related to the ancestral ones (H01–H10) have repeatedly vanished and re-emerged through recolonization. This group of populations, with H01 as the prevailing haplotype, dominated the scene for a reason beyond our comprehension. Being closely related to hypothetical ancestral haplotypes, it seems plausible that this group retained low levels of differentiation because of strong oscillations in its distribution range and abundance throughout evolutionary history. If a population periodically experiences severe genetic drift events, it is expected that emerging low-frequency mutations will constantly be purged^74^. Consequently, the inability of a lineage to accumulate new mutations will result in the constant presence of poorly differentiated haplotypes closely related to assumed ancestral ones. The absence of any substantial differentiation of these haplotypes across large areas can be additionally explained by rapid migrations and colonization that possibly occurred over long distances, which resulted in the absence of any significant divergence among remote sites^75^.

A comparison of haplotypes from the central and southern Apennines revealed that they were not as closely related as perhaps expected. For the most part, the central Apennines group harbours haplotypes found throughout the Balkans, with H01 being the predominant haplotype. At the same time, the group from the southern Apennines consisted of unique, more differentiated haplotypes not found anywhere else. It seems that Apennines groups of populations originated in two "out of Balkans" expansion events. From the first, more ancient colonization, the southern group emerged, while the second colonization event resulted in the formation of the central Apennines group of populations. Since the southern group haplotypes form a lineage that is exclusively found only in that region, it can be assumed that the colonization occurred just once and was followed by persistent isolation. The second colonization event likely occurred during the LGM. Since most of the haplotypes from the central Apennines are commonly found in the Balkans as well, there is no reason to think this group experienced a prolonged period of isolation. As already discussed, during the LGM, the land bridge between the Balkans and the Apennines was present and characterized by suitable environmental conditions that could easily support species range expansion.

Microsatellites and plastid DNA molecular markers allowed us to elucidate the evolutionary history of the common sage, not only during the recent periods but also more ancient ones. We proved again that the Apennines were colonized by the Balkans species, and in the case of the common sage, this occurred on two separate occasions. Although the results provided answers to numerous questions, they have also raised new ones. Rull^27^ stated that “microrefugia are no more than a theoretical necessity for explaining Pleistocene recolonization patterns”. However, it seems that there is also the possibility of microrefugia acting as a quarantine and not a pole position for recolonization. Regardless, although it was shown on more than several occasions that the “refugia within refugia” concept was applicable for explaining the present-day genetic structure of both plant and animal species in major refugium areas, this was not the case for the common sage.

## Methods

### Sampling and DNA extraction

**Figure 5:**
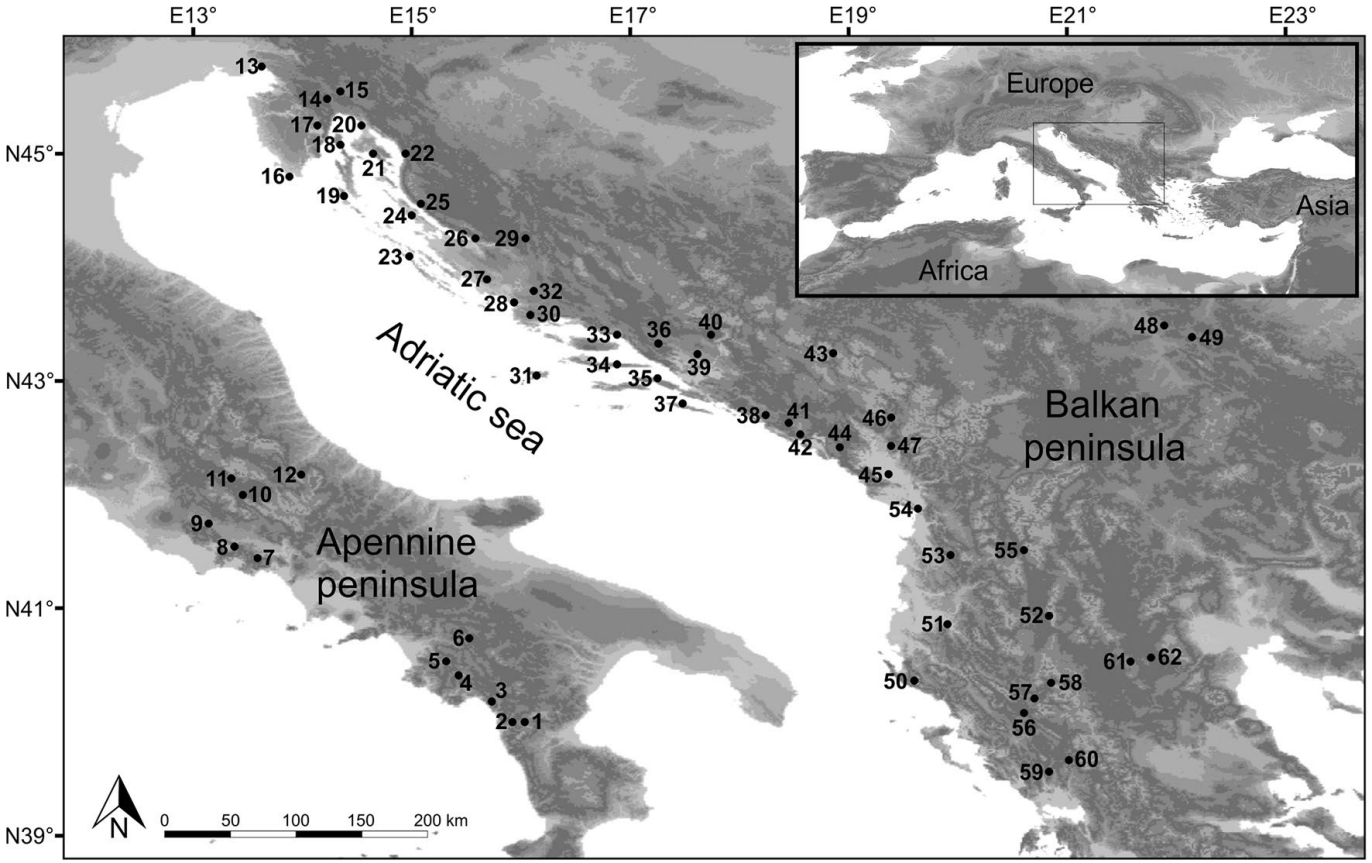
Sampling sites of the studied common sage (*Salvia officinalis* L.) populations. The satellite imagery was obtained from Natural Earth public domain map dataset (https://www.naturalearthdata.com/downloads/10m-raster-data/10m-gray-earth/). CorelDraw Graphics Suite X7 Version 17.1.0.572 (Corel Corp., Ottawa, Canada) was used to create the figure.

Sixty-two common sage populations distributed throughout the species distribution range were sampled (Fig. 5, Supplementary Table S5). The collection of plant material included in the research was carried out in accordance with relevant institutional, national, and international guidelines and legislation. The studied species is under “Least Concern” conservation status by the IUCN. Information on voucher specimens and who identified them is given in Supplementary Table S6.

For population genetic analysis, leaf material from 20 to 25 individuals from each population was collected and stored in silica gel for rapid desiccation. For the phylogeographic analysis, five samples were randomly chosen from each population sample set (310 samples in total). Genomic DNA was extracted from dried leaf material using the GenElute™ Plant Genomic DNA Miniprep Kit (Sigma–Aldrich^®^). After the extraction, the concentrations were measured on a spectrophotometer and the samples were diluted to 1 ng/μL for SSR and cpDNA analyses.

### Microsatellite amplification and data analysis

To assess the levels of genetic variability and structure of the sampled populations, eight previously characterized microsatellite markers were used (SoUZ001, SoUZ002, SoUZ003, SoUZ007, SoUZ011, SoUZ013, SoUZ014, and SoUZ019)^76,77^. Details of the polymerase chain reaction (PCR) conditions and the scoring of the amplified PCR products can be found in Radosavljević et al.^78^.

The average number of alleles per locus, the observed heterozygosity (*H*_*O*_) and the expected heterozygosity (*H*_*E*_) were calculated by Genepop v. 4.7^79^, while the allelic richness (*N*_*ar*_) and the private allelic richness (*N*_*par*_) of each population were estimated by HP-Rare v. 1.0^80^.

Genetic structure was inferred by a model-based clustering method implemented in STRUCTURE v. 2.3.4^81^. Details of the STRUCTURE analysis parameter set can be found in Radosavljević et al.^78^. Once the STRUCTURE results were obtained, the population genetic parameters generated earlier for the population-level analysis (*H*_O_, *H*_E_, *N*_ar_ and *N*_par_) were calculated for the established genetic clusters.

**Figure 6:**
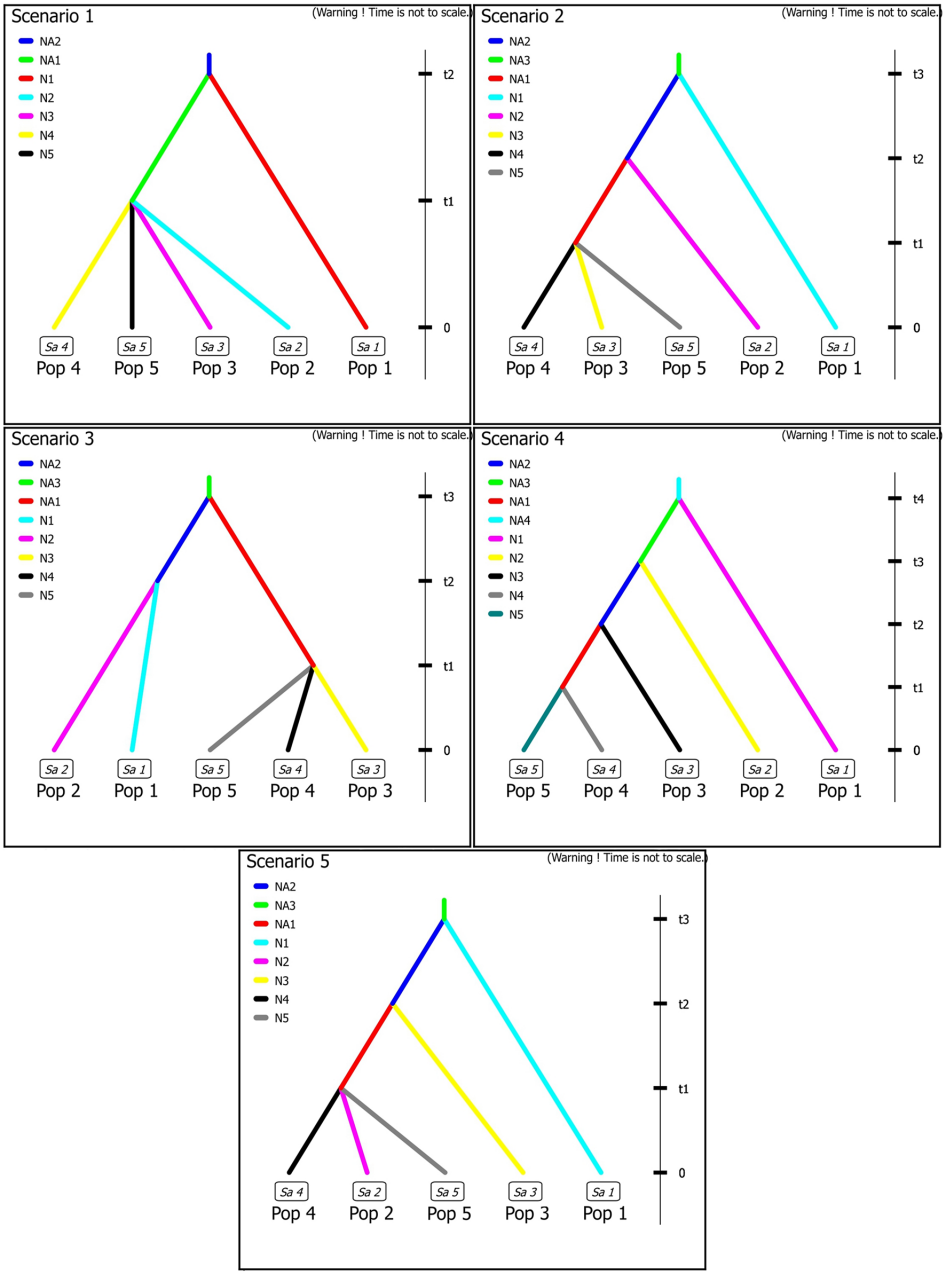
Graphical representation of tested historical scenarios of divergence among ancestral common sage (*Salvia officinalis* L.) populations in the Apennine and Balkan Peninsula using approximate Bayesian computation. t1–t4—times of past divergence events in terms of the number of generations, N1–N5 and NA1–NA4—effective population size during the given time period. Pop1–Pop5 correspond to SAp, CAp, NBalk, CBalk and SBalk, respectively.

To reconstruct the evolutionary history of divergence among ancestral populations, the ABC procedure (Approximated Bayesian Computation^82^), was performed using DIYABC v.2.0 software^83^. In accordance with the results obtained from the STRUCTURE analysis at K = 5, five populations were defined. In total, 41 sampled populations were selected for the ABC analysis, all characterized by a high proportion of membership of a specific ancestral population (Q > 0.7). Based on the obtained results, five competing scenarios were constructed (Fig. 6). (1) The population from the southern Apennines (in further text: SAp) diverged independently (at time t2), while the remaining present-day populations split from each other simultaneously sometime later (at t1). (2) The central Apennines population (CAp) diverged independently (at time t2), following earlier divergence of the SAp (at t3). Later, from the ancestral Balkan population, three remaining groups emerged (at t1). (3) The ancestral Apennines population diverged from the Balkan ancestral population (at t3). Later, Apennines populations diverged from each other (at t2), followed by the three-way split of the ancestral Balkan population (at t1). (4) Each population diverged independently. The earliest divergence is of the SAp (at t4), followed by the CAp (at t3) and population from the northern part of the western Balkans coastal region (NBalk) (t2). Finally, the population from the central part of the western Balkans coastal region and the southern Balkans population (CBalk and SBalk, respectively) split (at t1). (5) Following the initial divergence of SAp, the divergence of NBalk occurred. Later, from the remaining ancestral population, three present-day populations (i.e., CAp, CBalk, and SBalk) emerged. A detailed description of the run parameters is given in Supplementary Table S7, and the entire procedure (including software settings) was performed as described in Rešetnik et al.^46^.

### Chloroplast DNA sequence amplification and data analysis

To assess the phylogeographic structure of the studied species throughout its distribution area, two regions (*rps16-trnK* and *rpl32-trnL*) of the plastid genome were analysed. PCRs were performed in a total volume of 15 μL containing 1.5 μL 10 × PCR buffer (TaKaRa Taq™ Hot Start Version), 1.2 μL dNTP solution (2.5 mM each, TaKaRa Taq™ Hot Start Version), 0.6 μL 5 μM of each forward and reverse primer, 0.1 μL TaqHS polymerase (5 U/μL, TaKaRa Taq™ Hot Start Version) and 3 μL of 1 ng/μL DNA. The PCR conditions were 94 °C for 5 min, followed by 30 cycles of 95 °C for 1 min, 50 °C for 1 min and 65 °C for 4 min, and a final elongation step of 5 min at 65 °C. PCR products were cleaned using Exonuclease I (Fermentas, St. Leon-Rot, Germany) and FastAP Thermosensitive Alkaline Phosphatase (Fermentas, St. Leon-Rot, Germany) following the manufacturer’s instructions. Amplicons were sequenced in both directions using an ABI 3730XL analyser (Applied Biosystems).

DNA sequences were aligned using CLUSTAL X v. 2.1^84^ and manually edited where necessary in BioEdit v. 7.2^85^. Insertions or deletions (indels) in the cpDNA were treated as substitutions (single events)^86^.

Haplotype diversity within populations or groups of populations was quantified by calculating the number of haplotypes (*h*), the number of haplotypes per number of individuals (*h*/*n*), the number of private haplotypes (*h*_*pr*_), unbiased haplotype diversity (*H*_*d*_;^87^), and nucleotide diversity (π;^88^) using Arlequin v. 3.5.2.2^89^. Arlequin was also used to calculate the haplotypes distance matrix.

The median-joining haplotype network was constructed by PopART v. 1.7^90^ and manually redrawn in CorelDraw Graphics Suite X7 (Corel Corp., Ottawa, Canada) for better presentation.

The program Permut cpSSR v. 2.0^91,92^ was used to estimate genetic differentiation (*G*_*ST*_;^87,91^), considering only haplotype frequencies (unordered haplotypes), and the corresponding measure *N*_*ST*_^92^, considering genetic similarities between haplotypes (ordered haplotypes). A comparison of differentiation for ordered (*N*_*ST*_) vs. unordered (*G*_*ST*_) haplotypes was performed according to Pons and Petit^92^. Significance was tested based on 1000 random permutations. Phylogeographic structure is indicated when *N*_*ST*_ is higher than *G*_*ST*_ because closely related haplotypes are found more frequently in the same population than would be expected by chance.

Tajima's *D*^93^ and Fu's *F*_*S*_^94^ statistics were calculated using Arlequin to test for evidence of range expansion^95^. These parameters are very sensitive to deviations from population equilibrium. A significant value for *D* may be due to factors such as population expansion or bottlenecks^96,97^ and a significantly large negative value for *F*_*s*_ may be due to population expansion^94^. The significance of both test statistics was tested with 1,000 bootstrap replicates.

### Environmental niche modelling

To assess the geographical span of suitable environmental niches during the historical periods, environmental niche modelling (ENM) was performed as implemented in MAXENT ver. 3.3.3 k.^98^. The analysis was based on 62 occurrence records equally distributed throughout the species distribution range (locations of sampled populations included in the SSR analysis) following the procedure explained in Radosavljević et al.^78^ with all 19 WorldClim bioclimatic variables^99^ included. Retained bioclimatic variables and selected Maxent’s regularization multipliers were used to predict the potential distribution of the common sage for the present time, mid-Holocene (6000 YBP), LGM (21,000 YBP), and last interglacial (LIG, 120,000 YBP). For the LGM, three models were used: CCSM4, MIROC, and MPI-ESM-P. The obtained environmental suitability models were visualized in QGIS version 2.18.12. (QGIS Development Team (2020). QGIS Geographic Information System. Open Source Geospatial Foundation Project. http://qgis.osgeo.org).

## Supplementary Information

Supplementary Information.

## Data Availability

Sequences generated in this study are available in NCBI GenBank under accession numbers ON326101–ON326399 (rps16-trnK region) and ON325802–ON326100 (rpl32-trnL region).
