# Supplementary material for: Phylogeographic structure of common sage (Salvia officinalis L.) reveals microrefugia throughout the Balkans and colonizations of the Apennines
Source: Sci Rep. 2022 Sep 21;12:15726. doi: 10.1038/s41598-022-20055-4 (PMC9492721; doi:10.1038/s41598-022-20055-4)

Supplementary Information

Phylogeographic structure of common sage (Salvia officinalis L.) reveals microrefugia throughout the Balkans and colonizations of the Apennines

Ivan Radosavljević[1,2]*, Zlatko Satovic[2,3], Romeo di Pietro[4], Marija Jug Dujaković[5], Filip Varga[2,3], Danijel Škrtić[1], Zlatko Liber[1,2]

[1]University of Zagreb, Faculty of Science, Department of Biology, Division of Botany, Marulićev trg 9A, HR 10000 Zagreb, Croatia.
[2]Centre of Excellence for Biodiversity and Molecular Plant Breeding (CroP-BioDiv), Svetošimunska cesta 25, HR 10000 Zagreb, Croatia.
[3]University of Zagreb, Faculty of Agriculture, Department of Seed Science and Technology, Svetošimunska cesta 25, HR 10000 Zagreb, Croatia,
[4]Department PDTA (Section Environment and Landscape), "Sapienza" University of Rome, Via Flaminia 72, 00196 Rome, Italy
[5]Institute for Adriatic Crops and Karst Reclamation, Department for Plant Sciences, Put Duilova 11, 21000 Split, Croatia

* corresponding author

Table S1. Population-genetic parameters of the studied common sage (*Salvia officinalis* L.) populations.

Pop	genetic cluster/K5	n	N_{ar}	N_{pr}	N_{par}	H_O	H_E
P01	SAp	20	4.43	0	0.00	0.606	0.574
P02	SAp	23	4.42	0	0.00	0.522	0.519
P03	SAp	23	4.60	1	0.21	0.576	0.536
P04	SAp	24	4.52	1	0.08	0.516	0.521
P05	SAp	21	5.10	0	0.00	0.560	0.608
P06	SAp	22	4.29	1	0.12	0.591	0.591
P07	CAp	24	5.22	0	0.00	0.651	0.623
P08	CAp	20	5.46	0	0.01	0.606	0.628
P09	CAp	23	5.32	0	0.00	0.533	0.580
P10	CAp	20	3.41	0	0.05	0.531	0.500
P11	CAp	23	3.22	0	0.00	0.576	0.520
P12	CAp	20	1.35	0	0.00	0.081	0.087
P13	NBalk	24	5.53	0	0.00	0.630	0.650
P14	NBalk	24	3.27	0	0.00	0.474	0.527
P15	NBalk	21	5.65	0	0.00	0.750	0.717
P16	NBalk	23	6.66	0	0.01	0.701	0.695
P17	NBalk	19	6.16	0	0.07	0.697	0.718
P18	NBalk	21	6.99	0	0.02	0.762	0.733
P19	NBalk	22	7.14	1	0.11	0.682	0.700
P20	NBalk	23	7.56	0	0.09	0.717	0.739
P21	NBalk	22	7.29	0	0.04	0.790	0.760
P22	NBalk	21	7.28	0	0.12	0.667	0.772
P23	NBalk	23	7.44	0	0.04	0.669	0.678
P24	NBalk	24	7.86	0	0.19	0.755	0.761
P25	NBalk	24	6.85	0	0.13	0.724	0.773
P26	NBalk	24	7.45	0	0.05	0.714	0.736
P27	CBalk	23	7.04	0	0.09	0.712	0.708
P28	CBalk	16	9.13	1	0.25	0.719	0.759
P29	NBalk	21	7.49	0	0.23	0.691	0.749
P30	CBalk	25	8.49	0	0.01	0.735	0.785
P31	CBalk	20	6.42	0	0.27	0.713	0.711
P32	CBalk	22	8.80	1	0.15	0.733	0.779
P33	CBalk	18	7.87	0	0.04	0.750	0.763
P34	CBalk	21	9.18	1	0.26	0.774	0.775
P35	CBalk	24	9.70	0	0.34	0.745	0.768
P36	CBalk	24	8.83	1	0.18	0.734	0.781
P37	CBalk	22	8.47	1	0.19	0.665	0.767
P38	CBalk	25	10.10	0	0.17	0.825	0.846
P39	CBalk	23	9.33	0	0.25	0.832	0.817
P40	CBalk	19	9.54	0	0.18	0.757	0.805
P41	CBalk	19	9.89	1	0.48	0.809	0.807
P42	CBalk	19	8.45	0	0.13	0.750	0.775
P43	CBalk	20	6.01	1	0.45	0.625	0.606
P44	CBalk	23	7.61	0	0.10	0.832	0.806
P45	CBalk	20	9.16	0	0.22	0.819	0.814
P46	CBalk	20	8.74	1	0.34	0.775	0.823
P47	CBalk	22	8.42	1	0.20	0.676	0.767
P48	CBalk	17	4.43	0	0.00	0.463	0.508
P49	CBalk	20	5.03	0	0.02	0.644	0.614
P50	SBalk	20	8.48	5	0.85	0.744	0.777

P51	CBalk	24	5.20	0	0.02	0.557	0.648
P52	CBalk	24	4.50	0	0.01	0.578	0.537
P53	CBalk	24	6.70	0	0.05	0.651	0.734
P54	CBalk	22	8.59	0	0.08	0.705	0.794
P55	CBalk	19	5.69	1	0.39	0.612	0.644
P56	SBalk	20	7.34	0	0.07	0.631	0.701
P57	SBalk	24	5.97	0	0.03	0.557	0.636
P58	SBalk	24	5.56	0	0.09	0.708	0.683
P59	SBalk	24	4.99	0	0.09	0.578	0.595
P60	SBalk	21	5.90	4	0.74	0.708	0.655
P61	SBalk	20	6.54	1	0.25	0.663	0.703
P62	SBalk	23	5.43	0	0.13	0.674	0.667

genetic cluster/K5 - affiliation of studied *S. officinalis* populations with genetic clusters as recognized by STRUCTURE software at K = 5 as explained in the "Materials and methods" section. n – number of analysed individuals, N_{ar} – allelic richness, N_{pr} – number of private alleles, N_{par} – private allelic richness, H_O – observed heterozygosity, H_E – expected heterozygosity.

Table S2. Posterior probability of tested historical scenarios and 95% confidence intervals (CI) estimated with DIYABC analysis of five common sage (*Salvia officinalis* L.) populations

SCENARIO	Posterior probability	95% CI
1	0.4927	0.4768-0.5085
2	0.3152	0.3018-0.3287
3	0.1802	0.1700-0.1903
4	0.0057	0.0000-0.0140
5	0.0062	0.0000-0.0147

Table S3. ABC parameter estimation (median and 95% confidence intervals) for the best-supported scenario of the DIYABC analysis of five common sage (*Salvia officinalis* L.) populations

Parameter	Median	$Q_{2.5}$	$Q_{97.5}$
N1 (SAp)	3700	1170	9090
N2 (CAp)	945	208	5580
N3 (NBalk)	6270	2340	9560
N4 (CBalk)	9140	6590	9930
N5 (SBalk)	7430	3510	9760
t1	582	131	2060
NA1	27100	9260	70700
t2	7020	1330	64500
NA2	36100	8670	94200

N1 (SAp), N2 (CAp), N3 (NBalk), N4 (CBalk) and N5 (SBalk) – effective population sizes. t1 and t2 – time of the divergence events counted in generations, NA1 and NA2 – effective population sizes of ancestral populations, $Q_{2.5}$ – quantile 2.5%, $Q_{97.5}$ – quantile 97.5%.

Table S4. The distribution of detected haplotypes across studied populations of common sage (*Salvia officinalis* L.).

Pop	*n*	H01	H02	H03	H04	H05	H06	H07	H08	H09	H10	H11	H12	H13	H14	H15	H16	*h*	*h/n*
P01	5													5				1	0.20
P02	5											2		3				2	0.40
P03	5											1		4				2	0.40
P04	5												5					1	0.20
P05	5												5					1	0.20
P06	4												4					1	0.25
P07	5	4		1														2	0.40
P08	0																	0	0
P09	5	5																1	0.20
P10	5	3							2									2	0.40
P11	5	5																1	0.20
P12	4						4											1	0.25
P13	4	4																1	0.25
P14	5	5																1	0.20
P15	5					5												1	0.20
P16	5	1			4													2	0.40
P17	5	5																1	0.20
P18	5	1														4		2	0.40
P19	5	4			1													2	0.40
P20	5	5																1	0.20
P21	5	5																1	0.20
P22	5	4				1												2	0.40
P23	5	3		2														2	0.40
P24	5	5																1	0.20
P25	5															5		1	0.20
P26	5	5																1	0.20
P27	5	5																1	0.20
P28	5		3		1	1												3	0.60
P29	5	5																1	0.20
P30	5														1	4		2	0.40
P31	5	1			1	3												3	0.60
P32	5			5														1	0.20
P33	5	5																1	0.20
P34	5				5													1	0.20
P35	5			2						2							1	3	0.60
P36	5	2			3													2	0.40
P37	5			4													1	2	0.40
P38	5																5	1	0.20
P39	5	2		1	2													3	0.60
P40	5	3				2												2	0.40
P41	5					1											4	2	0.40
P42	5	1	4															2	0.40
P43	5	5																1	0.20
P44	5	5																1	0.20
P45	5																5	1	0.20

P46	5		2	3														2	0.40
P47	5		1	1												3		3	0.60
P48	5	5																1	0.20
P49	5	5																1	0.20
P50	5			5														1	0.20
P51	5															5		1	0.20
P52	4		4															1	0.25
P53	5		5															1	0.20
P54	5			2													3	2	0.40
P55	4	3									1							2	0.50
P56	5		5															1	0.20
P57	5															5		1	0.20
P58	5		5															1	0.20
P59	5															5		1	0.20
P60	5																5	1	0.20
P61	4		2					2										2	0.50
P62	5		4					1										2	0.40

H01-H16 – detected haplotypes, *n* – number of analysed individuals per population, *h* – number of detected haplotypes per population, *h/n* - number of haplotypes per number of individuals.

Table S5. Locations of sampled common sage (*Salvia officinalis* L.) populations and the number of samples used in SSR genotyping (n_{SSR}) and in chloroplast DNA sequencing (n_{cpDNA}).

Population	Country	Latitude (N)	Longitude (E)	Elevation m a.s.l.	n_{SSR}	n_{cpDNA}
P01	ITA	39.82	15.93	766	20	5
P02	ITA	39.82	15.97	996	23	5
P03	ITA	40.00	15.78	940	23	5
P04	ITA	40.23	15.45	841	24	5
P05	ITA	40.37	15.38	695	21	5
P06	ITA	40.57	15.55	656	22	4
P07	ITA	41.31	13.62	838	24	5
P08	ITA	41.40	13.36	815	23	0
P09	ITA	41.61	13.18	961	23	5
P10	ITA	41.88	13.49	1099	21	5
P11	ITA	42.01	13.41	900	23	5
P12	ITA	42.06	14.02	1100	20	4
P13	ITA	45.76	13.64	74	24	4
P14	SVN	45.49	14.30	960	22	5
P15	SVN	45.52	14.39	850	24	5
P16	HRV	44.77	13.91	20	25	5
P17	HRV	45.25	14.16	174	25	5
P18	HRV	45.06	14.37	202	24	5
P19	HRV	44.60	14.41	23	25	5
P20	HRV	45.23	14.57	103	25	5
P21	HRV	44.98	14.66	191	25	5
P22	HRV	44.98	14.98	591	24	5
P23	HRV	44.05	15.02	78	24	5
P24	HRV	44.43	15.04	129	25	5
P25	HRV	44.52	15.10	167	24	5
P26	HRV	44.20	15.62	152	25	5
P27	HRV	43.83	15.72	99	24	5
P28	HRV	43.63	15.96	8	24	5
P29	HRV	44.21	16.06	330	24	5
P30	HRV	43.51	16.12	8	25	5
P31	HRV	43.03	16.14	201	24	5
P32	HRV	43.73	16.16	325	25	5
P33	HRV	43.40	16.92	432	24	5
P34	HRV	43.13	16.95	334	25	5
P35	HRV	42.98	17.27	483	25	5
P36	HRV	43.36	17.27	394	24	5
P37	HRV	42.75	17.51	325	25	5
P38	HRV	42.60	18.25	553	25	5
P39	BIH	43.18	17.69	216	25	5
P40	BIH	43.33	17.75	367	25	5

P41	MNE	42.53	18.49	646	19	5
P42	MNE	42.41	18.57	179	19	5
P43	MNE	43.17	18.86	756	20	5
P44	MNE	42.30	18.92	850	23	5
P45	MNE	42.06	19.37	257	20	5
P46	MNE	42.57	19.39	570	20	5
P47	MNE	42.32	19.41	8	22	5
P48	SRB	43.43	21.87	309	17	5
P49	SRB	43.31	22.12	504	20	5
P50	ALB	40.18	19.60	424	22	5
P51	ALB	40.70	19.90	180	19	5
P52	ALB	40.77	20.83	1164	24	4
P53	ALB	41.33	19.94	842	24	5
P54	ALB	41.79	19.63	30	24	5
P55	MKD	41.37	20.60	617	20	4
P56	GRC	39.94	20.67	598	20	5
P57	GRC	40.03	20.73	383	24	5
P58	GRC	40.14	20.84	649	24	5
P59	GRC	39.39	20.87	197	24	5
P60	GRC	39.46	21.02	599	21	5
P61	GRC	40.32	21.65	873	22	4
P62	GRC	40.33	21.71	714	24	5

Table S6. Voucher information of 62 sampled common sage (*Salvia officinalis* L.) populations.

Pop	Locality	Voucher ID	Identified by
P01	Pollino 2	HFLA 5020	Romeo di Pietro
P02	Pollino 1	HFLA 5021	Romeo di Pietro
P03	Maratea	HFLA 5022	Romeo di Pietro
P04	Cilento	HFLA 5023	Romeo di Pietro
P05	Sacco	HFLA 5024	Romeo di Pietro
P06	Savoia	HFLA 5025	Romeo di Pietro
P07	Aurunci	ZA 33412	Romeo di Pietro
P08	Ausoni	ZA 33413	Romeo di Pietro
P09	Supino	HFLA 5026	Romeo di Pietro
P10	d'Antino	HFLA 5027	Romeo di Pietro
P11	M. Salviano	HFLA 5028	Romeo di Pietro
P12	Maiella/Pacentro	HFLA 5029	Romeo di Pietro
P13	Trieste	ZA 33415	Romeo di Pietro
P14	Novokračine	NHMR 2195	Boštjan Surina
P15	Ilirska Bistrica	ZA 33414	Boštjan Surina
P16	Kamenjak	ZA 26313	Marija Jug Dujaković
P17	Šušnjevica	ZA 26304	Marija Jug Dujaković
P18	Cres	ZA 26295	Marija Jug Dujaković
P19	Lošinj	ZA 26303	Marija Jug Dujaković
P20	Krk	ZA 26309	Marija Jug Dujaković
P21	Stara Baška	ZA 26297	Marija Jug Dujaković
P22	Vratnik	ZA 26300	Marija Jug Dujaković
P23	Dugi Otok	ZA 26301	Marija Jug Dujaković
P24	Pag	ZA 26314	Marija Jug Dujaković
P25	Karlobag	ZA 26310	Marija Jug Dujaković
P26	Otišina	ZA 26307	Marija Jug Dujaković
P27	Pirovac	ZA 26311	Marija Jug Dujaković
P28	Šparadići	ZA 26302	Marija Jug Dujaković
P29	Zrmanja	ZA 26293	Marija Jug Dujaković
P30	Vinišće	ZA 26315	Marija Jug Dujaković
P31	Vis	ZA 26299	Marija Jug Dujaković
P32	Unešić	ZA 26316	Marija Jug Dujaković
P33	Biokovo	ZA 26296	Marija Jug Dujaković
P34	Hvar	ZA 26294	Marija Jug Dujaković
P35	Pelješac	ZA 26298	Marija Jug Dujaković
P36	Runovici	ZA 26305	Marija Jug Dujaković
P37	Mljet	ZA 26308	Marija Jug Dujaković
P38	Konavle	ZA 26312	Marija Jug Dujaković
P39	Međugorje	ZA 26292	Marija Jug Dujaković
P40	Mostar	ZA 26306	Marija Jug Dujaković
P41	Vrbanj	NHMR 3165	Marija Jug Dujaković
P42	Luštica	NHMR 3166	Marija Jug Dujaković
P43	Piva	NHMR 3167	Marija Jug Dujaković
P44	Brajići	ZA 72805	Marija Jug Dujaković
P45	Rumija	NHMR 3168	Marija Jug Dujaković
P46	Bratonožići	NHMR 3169	Marija Jug Dujaković
P47	Božaj	ZA 72806	Marija Jug Dujaković

P48	Miljkovac	NHMR 3170	Zlatko Satovic
P49	Sićevo	NHMR 3171	Zlatko Satovic
P50	Llogora	AI005	Marija Jug Dujaković
P51	Tommor	ZA 72807	Marija Jug Dujaković
P52	Prespa	ZA 72808	Marija Jug Dujaković
P53	Dajti	ZA 72809	Marija Jug Dujaković
P54	Shengjin	ZA 37621	Marija Jug Dujaković
P55	Jablanica	MKDSo14/09	Marija Jug Dujaković
P56	Zagori	ZA 72810	Marija Jug Dujaković
P57	Timfi	ZA 37624	Marija Jug Dujaković
P58	Smolikas	ZA 37623	Marija Jug Dujaković
P59	Arta	ZA 37622	Marija Jug Dujaković
P60	Plaka	ZA 72811	Marija Jug Dujaković
P61	Skiti, Kozani	13/2010*	Marija Jug Dujaković
P62	Lygeri, Kozani	12/2010*	Marija Jug Dujaković

HFLA - Herbarium Flaminio of Sapienza University of Rome, Rome, Italy; ZA - Herbarium Croaticum, University of Zagreb, Faculty of Science, Zagreb, Croatia; NHMR - Herbarium of Natural History Museum Rijeka, Rijeka, Croatia; AI - Faculty of Agriculture and Environment, Agricultural University of Tirana, Tirana, Albania; MKD - Institute of Pharmacognosy, Faculty of Pharmacy, Skopje, Republic of Macedonia; * - Department of Aromatic and Medicinal Plants, Hellenic Agricultural Organisation, Thessaloniki, Greece.

Table S7. Parameters used in DIY ABC analysis of five common sage (*Salvia officinalis* L.) populations.

Parameter	Conditions	Distribution (min – max)
N1	-	Uniform (10 – 10000)
N2	-	Uniform (10 – 10000)
N3	-	Uniform (10 – 10000)
N4	-	Uniform (10 – 10000)
N5	-	Uniform (10 – 10000)
NA1	NA1>N2, NA1>N3, NA1>N4, NA1>N5	Uniform (10 – 100000)
NA2	NA2>NA1, NA2>N1, NA2>N2, NA2>N3	Uniform (10 – 100000)
NA3	NA3>NA1, NA3>NA2, NA3>N1, NA3>N2	Uniform (10 – 100000)
NA4	NA4>NA3, NA4>N1	Uniform (10 – 100000)
t1	-	Uniform (1 – 100000)
t2	t2>t1	Uniform (1 – 100000)
t3	t3>t1, t3>t2	Uniform (1 – 100000)
t4	t4>t1, t4>t2, t4>t3	Uniform (1 – 100000)
Mean mutation rate	-	Uniform (10^{-4} – 10^{-3})
Individual locus mutation rate	-	Gamma (10^{-5} – 10^{-2})
Mean coefficient P	-	Uniform (10^{-1} – $3x10^{-1}$)
Individual locus coefficient P	-	Gamma (10^{-2} – $9x10^{-1}$)
Mean SNI rate	-	Log-u (10^{-8} – 10^{-5})
Individual locus SNI rate	-	Gamma (10^{-9} – 10^{-4})

t1-t4 - times of past divergence events in terms of the number of generations, N1-N5 and NA1-NA4 - effective population size during the given time period.

Figure S1. Principal Component Analysis (PCA) obtained by DIYABC.

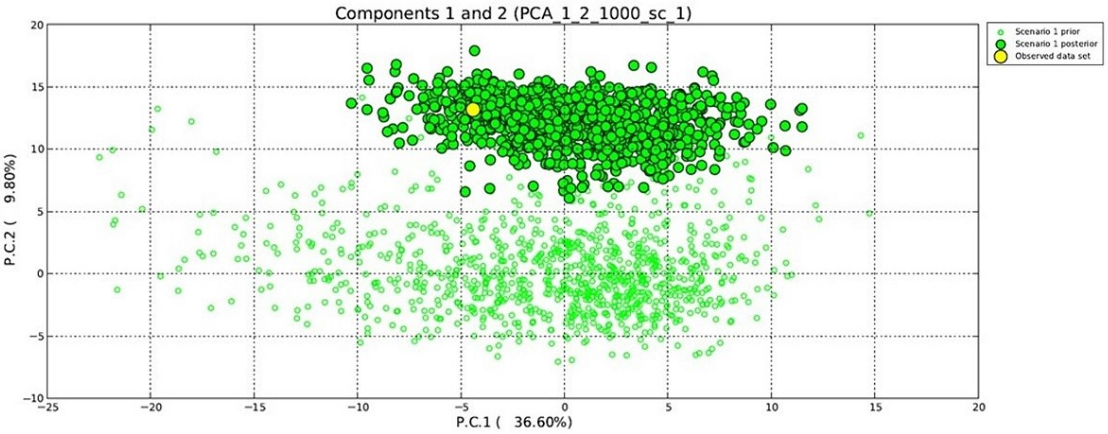

Supplement: Supplementary file 1 — Supplementary Information. [file 41598_2022_20055_MOESM1_ESM.pdf]
